# Receptor tyrosine kinase (RTK) targeting in pediatric high-grade glioma and diffuse midline glioma: Pre-clinical models and precision medicine

**DOI:** 10.3389/fonc.2022.922928

**Published:** 2022-08-01

**Authors:** Kallen Schwark, Dana Messinger, Jessica R. Cummings, Joshua Bradin, Abed Kawakibi, Clarissa M. Babila, Samantha Lyons, Sunjong Ji, Rodrigo T. Cartaxo, Seongbae Kong, Evan Cantor, Carl Koschmann, Viveka Nand Yadav

**Affiliations:** ^1^ Department of Pediatrics, Division of Pediatric Hematology-Oncology, University of Michigan School of Medicine, Ann Arbor, MI, United States; ^2^ Department of Pediatrics, Children's Mercy Research Institute (CMRI), Kansas, MO, United States; ^3^ Department of Pediatrics, University of Missouri Kansas City School of Medicine, Kansas, MO, United States

**Keywords:** glioma, TKI - tyrosine kinase inhibitor, RTK - receptor tyrosine kinase, pediatric, neuro-oncology - medical, high-grade glioma (HGG), preclinical (*in vivo*) studies, mouse models

## Abstract

Pediatric high-grade glioma (pHGG), including both diffuse midline glioma (DMG) and non-midline tumors, continues to be one of the deadliest oncologic diagnoses (both henceforth referred to as “pHGG”). Targeted therapy options aimed at key oncogenic receptor tyrosine kinase (RTK) drivers using small-molecule RTK inhibitors has been extensively studied, but the absence of proper *in vivo* modeling that recapitulate pHGG biology has historically been a research challenge. Thankfully, there have been many recent advances in animal modeling, including Cre-inducible transgenic models, as well as intra-uterine electroporation (IUE) models, which closely recapitulate the salient features of human pHGG tumors. Over 20% of pHGG have been found in sequencing studies to have alterations in platelet derived growth factor-alpha (PDGFRA), making growth factor modeling and inhibition *via* targeted tyrosine kinases a rich vein of interest. With commonly found alterations in other growth factors, including FGFR, EGFR, VEGFR as well as RET, MET, and ALK, it is necessary to model those receptors, as well. Here we review the recent advances in murine modeling and precision targeting of the most important RTKs in their clinical context. We additionally provide a review of current work in the field with several small molecule RTK inhibitors used in pre-clinical or clinical settings for treatment of pHGG.

## Introduction

Pediatric high-grade gliomas (pHGGs) are one of the leading causes of cancer-related death in children. pHGGs are further divided into multiple subgroups based on tumor location and mutation status. pHGGs encompass both hemispheric (non-midline) and diffuse midline gliomas (DMGs) and represent the most lethal pediatric brain tumors. Radiation remains the only proven therapy to extend survival to date and less than 20% of patients survive for five years past diagnosis (1). pHGG was identified as early as 1926; for several decades, its biological behavior was believed to be like adult high-grade gliomas and was treated with similar therapeutic regimens (2). These treatments failed to improve patient survival, which led researchers to investigate if pHGG has a biologically distinct phenotype from adult gliomas (3).

To combat the dismal prognosis for these patients, molecular drivers for this tumor subset are being investigated using sequencing and preclinical models. Several groups used high-throughput whole-genome, whole-exome, and transcriptome sequencing to characterize mutations in pediatric gliomas and differences from adult gliomas (4–6). Wu et al. identified that ~80% DMG contain a somatic point mutation in the *H3K27M* gene and 16% of cortical pHGG contain H3.3 G34R/V mutations (7). *H3K27M* mutations occur frequently in *H3F3A* and to a lesser extent in *H3C2* encoding H3.1 (25% of cases), while *H3.3G34R/V* mutations occur exclusively in *H3F3A*. These onco-histone mutations in pHGG have been recognized as examples of key genetic drivers that play an integral role in tumor development (8–10). Pediatric gliomas have been found to have fewer drivers than their adult counterparts, which suggests that identifying the drivers that do exist *via* sequencing will lead to effective strategies for precision therapeutics (11).

**Figure 1:**
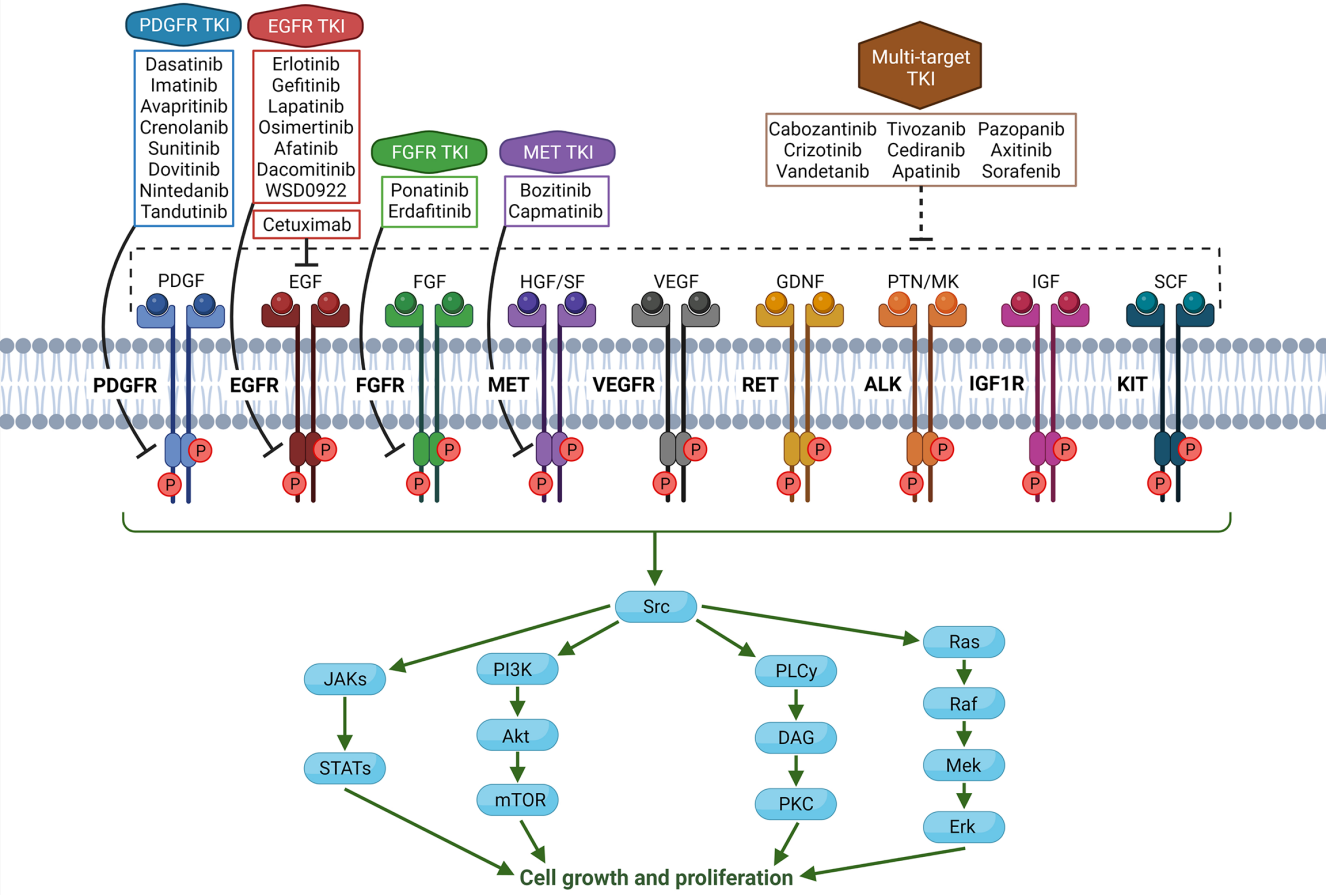
Schema of common and targetable receptor tyrosine kinases (RTKs) in the human body, along with associated tyrosine kinase inhibitors (TKIs). In glioma cells, overexpression, mutation, or amplification of RTKs can lead to tumorigenic phenotype. Note that all TKIs have varying degrees of off-target effects on other RTKs or kinases in the cell, and TKIs being specific to one receptor is an oversimplification. (Created with Biorender.com).

Apart from histone mutations, the most common alterations in pHGG are overexpression of receptor tyrosine kinase (RTK) family members. Paugh et al. showed that most pHGGs demonstrated amplification of platelet-derived growth factor receptor A (PDGFRA)-driven signal, while adult HGG shows EGFR overexpression (6). Amplification of *PDGFRA* commonly leads to activation of the PI3K/mTOR or MAPK signaling pathways in pHGG (3, 12, 13). Alterations in *PDGFRA* are significant, as they are associated with worse prognoses (14). Many tyrosine kinase inhibitors (TKIs) have been developed as targeted therapies for cancers with similar RTK alterations, and research on their efficacy in pHGG is ongoing. A schematic of RTKs and TKIs that have been trialed in adult or pediatric brain tumors is shown in **Figure 1**.

**Figure 2:**
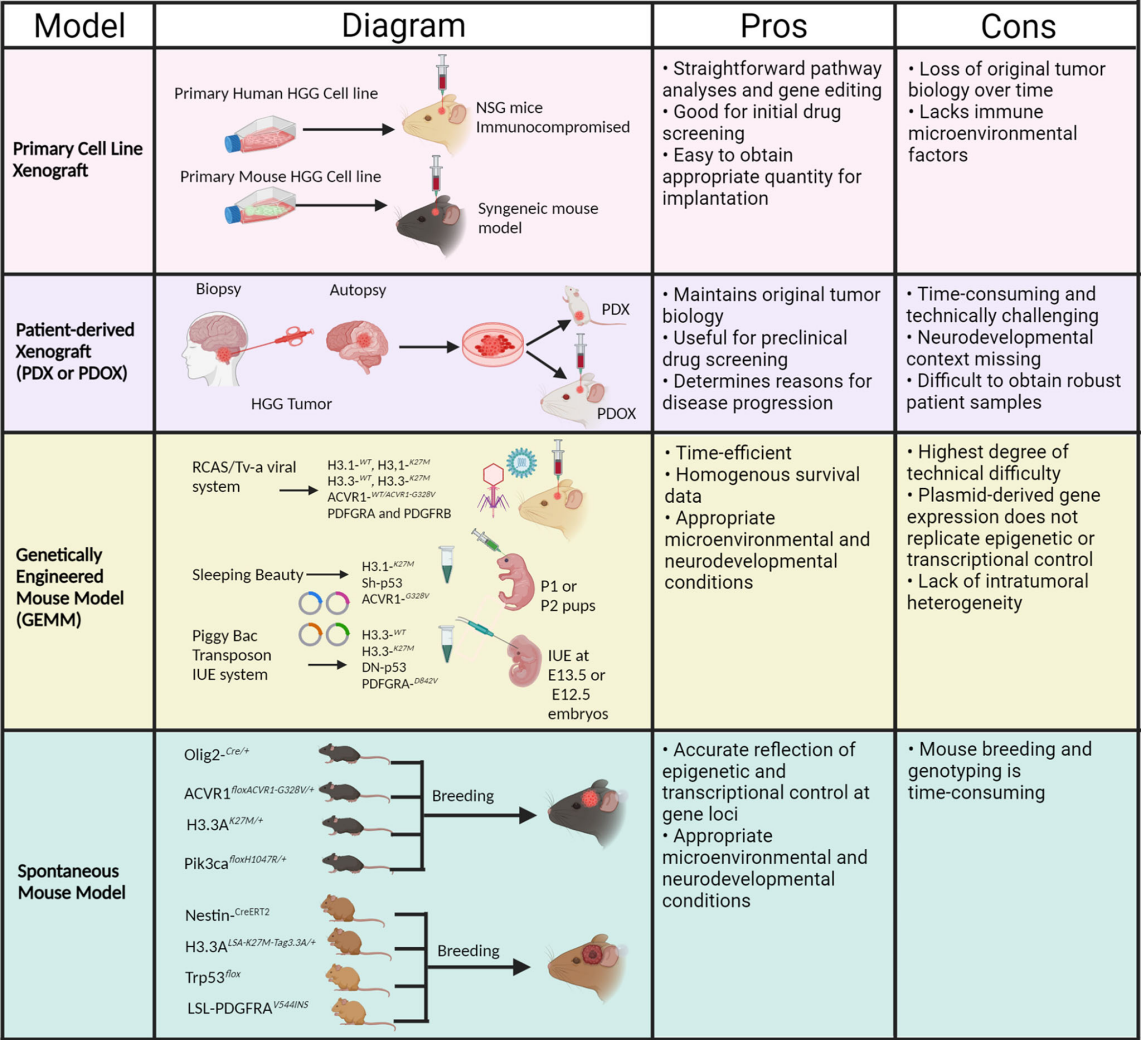
Schema of preclinical mouse models and associated pros and cons for pHGG. (Created with Biorender.com).

Understanding molecular mechanisms of specific tyrosine kinase alterations and their therapeutic impact on pHGG tumorigenesis warrants accurate genetic mouse models, which can recapitulate the salient features of pHGG for precise preclinical testing. Multiple models have been developed for the identification of potential therapeutics against pHGG, such as cell line xenografts and genetically engineered mouse models (15). Using these models, therapies that target these alterations can be developed and potentially lead to effective treatment strategies for pediatric gliomas. In this review, we outline our current understanding of RTK alterations in pediatric high-grade gliomas, as well as the status of their corresponding preclinical models and possible treatment methods. Illustrative description of all preclinical models of pHGG are depicted in **Figure 2**.

## Landscape of RTK alterations

RTK pathway alterations are common in pediatric glioma. Wu et al. sequenced 127 pediatric HGGs and found RTK-RAS-PI3K signaling pathway alterations in 69% of diffuse intrinsic pontine gliomas (DIPGs) and 67% of non-brainstem-HGGs (NBS-HGGs) (7). *PDGFRA* alterations were found in 20% of DIPGs and 21% of NBS-HGGs, and *EGFR* alterations were found in 10% of NBS-HGGs (none in DIPGs). These alterations were mostly amplifications, with some indel mutations, missense mutations, and structural variants. Mackay et al. obtained copy number profiles from 834 cases of pHGGs and found 77 *PDGFRA* amplifications (9%), 32 *EGFR* amplifications (4%), and 19 *MET* amplifications (2%). They also analyzed sequencing data from 326 pHGG samples and found 19 *PDGFRA* mutations (6%), 11 *EGFR* mutations (3%), 12 *FGFR1* or *FGFR2* mutations (4%), 3 *MET* mutations (1%), and 1 *IGF1R* mutation (0.3%) (16). Guerriero Stucklin et al. performed a large-scale analysis of infantile hemispheric glioma and identified one subgroup of tumors that harbored alterations in *ROS1* (21%), *NTRK* (21%), *ALK* (15%), and *MET* (6%) (17).

These alterations are significant because they change patient prognosis. In integrated sequencing datasets from 290 pediatric HGG patients, *PDGFRA* amplification significantly reduced overall survival in non-brainstem HGG when compared to patients without the amplification (14). Recently, Mondal et al. found pediatric bithalamic midline glial tumors that lacked the H3K27M mutation but harbored frequent insertion mutations in exon 20 or frameshift mutations in exon 7 of the *EGFR* oncogene (mutations - 11/13, 85%; amplifications – 1/13, 7.7%) that confer sensitivity to specific TKIs (osimertinib and afatinib) *in vitro* (13). Soon after, DNA methylation profiling was done to identify a group of pHGGs that were epigenetically similar to five of the bithalamic gliomas from Mondal et al.; this group was mostly composed of midline gliomas and frequently carried *EGFR* alterations (mutations – 20/30, 67%; amplifications – 16/58, 27%) (18). This contrasts with non-midline cortical pHGG, which do not commonly carry *EGFR* alterations (7); and is similar to adult glioblastoma (GBM), with as many as 60% of GBMs carrying *EGFR* amplification (19). With the optimization of patient-specific molecular profiling for high-grade brain tumors like pHGG, personalized therapeutics will be critical for effective treatments. To this end, developing personalized animal models with specific genetic lesions present in pHGG will be an important step in optimizing therapeutics in a preclinical setting.

## PDGFRA-driven pHGG modeling *in vitro*

Because *PDGFRA* is frequently amplified or mutated in pediatric gliomas, there is a need to replicate different *PDGFRA* variant types found in pHGG *via* modeling (6, 20). Paugh et al. introduced six *PDGFRA* activating mutants affecting different parts of the receptor, along with wildtype, into neural precursor cells (NPCs) cultured *in vitro* using retroviral constructs. They observed that *PDGFRA* mutants are constitutively active and have a proliferative advantage over empty vector controls in *TP53*-null primary mouse astrocytes (PMAs) (21). Further, Funato et al. showed that expression of *PDGFRA-D842V* synergizes with *H3.3K27M* and *TP53* loss in NPCs, resulting in de-differentiation and neoplastic transformation (22).

*In vitro PDGFRA-*amplified models have also been generated through overexpressing wild-type *PDGFRA*. Pathania et al. found that *in vitro* cells from murine gliomas harboring *H3.3K27M, TP53* knockdown, and *PDGFRA-*WT overexpression were more invasive than isogenic cells without induced *PDGFRA* amplification (21). Cells with *PDGFRA-*WT overexpression did not display receptor activation when in a ligand-free setting; however, when treated with PDGF-AA ligand, these cells had increased receptor activation to even higher levels than *PDGFRA* mutant cells. This suggested a possible increase in ligand dependence in *PDGFRA-*amplified variants compared to mutated variants (21).

## Patient-derived xenograft models

Patient-derived xenograft (PDX) models provide a mechanism to study biologically relevant tumors in an *in vivo* setting. Coupled with anatomically relevant sites and tumors that maintain their original biology, PDX models can accurately recapitulate HGG and provide insight into the factors behind disease progression (23). These models are particularly valuable for preclinical screening of therapeutic agents and may be useful as a prognostic factor for many cancers (24).

Historically, because pHGG was typically diagnosed through features observed on imaging studies rather than surgical biopsies, there was a deficiency of the readily available tissue resources often used for preclinical therapeutic testing (25). In addition, growing tumors in the appropriate location *in vivo* has been challenging. Advances in xenograft technology have since allowed the establishment of anatomically accurate mouse models that have been used to examine patterns of growth and response to novel therapeutic agents in pHGG. These human derived glioma models retain the invasive patterns of growth and the morphological and pathological characteristics of the parental tumor (26). Furthermore, Brabetz et al. showed that PDX models show a differential response to therapy based on molecular drivers such as *EGFR* amplification (27).

Recently, He et al. established 21 PDX models for pHGG and verified their accurate recapitulation of tumor histopathology, mutations, and gene expression patterns. They tested a panel of 1134 FDA-approved drugs, including TKIs such as afatinib, ponatinib, and dasatinib, in pHGG cell lines derived directly from the xenograft models (28). Ultimately, they investigated dual therapy with PI3K/mTOR and MEK inhibitors, finding evidence of synergistic growth inhibition *in vitro* and significant survival extension *in vivo*. In summary, patient-derived xenografts allow for improved preclinical testing of new therapeutic targets for pHGG in a tumor- and organ-specific manner (28). However, PDX models do not recreate the brain developmental context and the impact of the immune microenvironment on tumor growth, leading the field to investigate genetically engineered models.

## Genetically engineered mouse models (GEMM)

### RCAS model system

The replication-competent avian sarcoma-leucosis virus (RCAS)-TVA system is a genetically engineered mouse model that can be used to create tumors containing specific drivers of gliomagenesis, such as PDGF. The virus is modified to contain the genes to be inserted into the host genome, and TVA is the receptor for viral entry. Brain cells are specifically engineered to express the TVA receptor, allowing targeted gene transfer (29).

Using this model, Hambardzumyan et al. showed that Ntv-a mice overexpressing *PDGFB* can form tumors in multiple brain regions but form higher-grade tumors with increased efficiency when the mice also lack the Ink4a-ARF tumor suppressors (30). Barton et al. used the RCAS PDGF-B; Ink4a-ARF deficient mouse model to test the effects of the CDK4/6 inhibitor PD-0332991 (PD) on brainstem gliomas (31). More recently, Hoeman et al. introduced *ACVR1* mutations into brainstem progenitor cells *via* RCAS and determined the R206H mutation works alongside H3.1K27M in promoting gliomagenesis (32). Finally, Halvorson et al. used cells derived from their RCAS mouse models for pHGG and identified the TKI BMS-754807 as a potent inhibitor of proliferation in tumor-derived cells (33). In summary, the RCAS-TVA system is a reliable method for creating pHGG models with specific tumor drivers and can be used for testing therapeutic efficacy of TKIs.

### Sleeping Beauty (SB)

The Sleeping Beauty (SB) model is a DNA transposon system that has been used to induce HGGs in mice with specific genetic lesions. In this system, the SB transposase recognizes inverted repeat and direct repeat (IR/DR) sequences flanking the transposons and facilitates their stable integration into the host genome *via* a “cut and paste” mechanism. The SB method involves transfecting subventricular zone NPCs into 1-day-old mouse pups by injecting a combination of SB DNA transposon plasmids bearing mutations specific to pHGG. Different combinations of these plasmids have been used to generate highly penetrant gliomas using the SB model, including common mutations seen in HGG such as mutant *H3.3K27M, ATRX, ACVR1-G328V*, and *IDH1*, along with tumor drivers NRAS and shRNA KO *TP53* (shp53) (34–36).

The SB model has been recently used to study the impact of the tumor microenvironment, specifically the role of myeloid cell immunosuppression on mutant IDH1 tumor growth (37). Recently, Mendez et al. used this model to develop a murine brainstem pHGG tumor using *H3K27M* and *ACVR1-G328V* genetic alterations, along with NRAS and shp53 as oncogenic drivers (38). SB has also been used to study RTK activity in gliomas; Qin et al. injected SB plasmids with *HGF* and *MET* cDNA as well as shRNA against p53 into lateral ventricles of neonatal mice and saw *MET*-driven glioma development (39). They tested two MET inhibitors, V-4084 and SGX523, and found sensitivity to both in isolated neurosphere cell lines. The SB model tumors arise *de novo* and recapitulate the human disease at both histological and molecular levels, providing a venue for testing targeted therapies in tumors with specific RTK drivers.

### Intrauterine electroporation (IUE)

Studying the molecular role of growth factors (e.g., *PDGFRA*) in pHGG pathogenesis warrants accurate genetic and epigenetic models that retain features of pHGG. Recently, the *in-utero* electroporation (IUE) method has been used to generate pHGG models through transfecting NPCs of prenatal mice on embryonic day 12.5-13.5 (E12.5-13.5) using PiggyBac DNA transposon plasmids bearing characteristic mutations of pHGG (40, 41). This model reproduces the neurodevelopmental attributes of pHGGs in younger patients (i.e., tumor formation in a developing brain) and generates infiltrative *de novo* tumors within the developing mouse brain that are histologically consistent with human pHGG. Varying combinations of these plasmids have been used to generate highly penetrant gliomas throughout the evolution of this model, including mutations common in pHGG such as mutant *H3.3K27M*, *ATRX*, *PDGFRA-D842V*, and *TP53*.

Naturally, the IUE method requires significant precision in timing and gene specificity. Pathania et al. targeted NPCs in the lower rhombic lip of the developing hindbrain of E12.5 mice and found that *H3.3K27M* mutation and *TP53* loss are sufficient to induce diffuse tumors characteristic of human pHGG in both hindbrain and forebrain regions after 6-8 months. In addition to these genes, they determined that including *ATRX* knockdown *via* shRNA resulted in more circumscribed tumors after 4 months, and further addition of *PDGFRA* to the IUE model shortened the period of tumor growth to 21 days (21). In contrast, *H3.3K27M* and *TP53* loss did not result in tumorigenesis in a later study performed by Patel et al. at E13.5 in the hindbrain, suggesting an important developmental window between E12-14 for tumorigenesis with this specific combination. They also found that *PDGFRA-D842V* activating mutation and *TP53* loss were able to cause malignant transformation without *H3.3K27M* mutation (40).

Combinations of *PDGFRA-D842V, PDGFRA^-^WT*, or *PDGFB* along with *DNp53* and H3.3K27M have resulted in tumors with varying histological and molecular features seen in brainstem pHGG, suggesting that the IUE method can be used to learn about the pathological heterogeneity associated with oncogenic drivers of pHGG. Patel et al. found that *PDGFB-*induced tumors formed rapidly and displayed vascular remodeling and angiogenesis, while *PDGFRA-D842V*-induced tumors were highly invasive with minimal vascular abnormalities (40). Integration of *PDGFRA^-^WT* produced both high- and low-grade gliomas with extended latencies.

Recently, our group used IUE to generate murine pHGG models with plasmids expressing *dnTP53, H3.3K27M* and *PDGFRA-D842V* mutations, leading to highly invasive HGGs in the forebrains of mice (41). Total upregulation of *PDGFRA* was confirmed *via* IHC, and this pathway was targeted with co-administration of the TKI dasatinib and mTOR inhibitor everolimus. Co-treatment significantly extended the survival of tumor-bearing mice beyond that of dasatinib treatment, providing evidence for a potential improvement in targeted therapy for *PDGFRA*-altered tumors. As seen from these results, the IUE model can be instrumental in providing an effective model that closely mimics human tumor development for targeting RTKs in pHGG (41).

### Spontaneous GEMM

Genetically engineering a mouse model that endogenously expresses an activating mutation in PDGFRA helps define the role this mutation plays in spontaneous tumorigenesis. Specifically, use of a Cre-inducible system allows the effects of mutant *PDGFRA* to be studied solely in the brain (42). The LSL-*PDGFRA-V544ins* transgenic mouse model expresses a heterozygous duplication in a short segment of the transmembrane domain and is induced in mice shortly after birth to model pediatric disease. When combined with a *TP53* knockout mutation (TP53 cKO), mice developed HGG brainstem tumors that closely mimicked traits of human DIPG (brain infiltration, variable astrocyte differentiation, nuclear staining of Olig2); in addition, the cells robustly expressed *PDGFRA* in the cytoplasm (42). These data indicate that *PDGFRA* activating mutations alone are not enough to drive tumorigenesis but will cooperate with knockout of *TP53* to direct formation of HGG within the brainstem, significantly decreasing survival.

Similarly, Fortin et al. developed spontaneous midbrain and thalamic HGG models by knocking in H3.1K27M, ACVR1-G328V, and PIK3CA-H1047R. The respective endogenous loci were driven by Cre recombinase in Olig2-positive oligodendrocyte precursor cells (OPCs) (43). Zou et al. used Cre recombinase to knock in mutant *PDGFRA* combined with Ink4a/ARF -/- in OPCs of mice to cause development of brain tumors resembling anaplastic human gliomas, emphasizing the importance of *PDGFRA* as an early driver of malignant transformation of OPCs (44). This and similar models made with Cre recombinase would provide an accurate model for testing the therapeutic effects of TKIs in RTK-altered pHGG.

## Comparison of pHGG preclinical models

### pHGG cell line and patient-derived xenografts

With the recent advances in surgical techniques, several groups have developed many pHGG cell lines using human tumor cells directly from biopsy and autopsy samples. These cell lines are very effective for *in vitro* studies, including drug screening, molecular pathway analysis, and specific gene editing. However, one major caveat is the fidelity of these cell lines compared to the actual tumor tissue. When cells are continuously passaged in artificial media, extensive clonal selection with high mutation frequency occurs. Recently, Filbin et al. compared the gene profiling of DIPG cell lines with the original corresponding tumor using single cell RNA sequencing analysis and found stark differences in expression (45). Of their models tested, tumor maintenance *via* PDX in mice was most accurate to the original tumor biology, followed by primary sphere cell lines, and the least accurate were adherent glioma cell line models.

Unlike xenografts from cell lines, PDXs have shown strong genetic and transcriptomic correlation with the original tumor when characterized, as well as retained histological features of the original tumor when implanted into immunodeficient mice (27, 46). However, PDX models are technically challenging and can take up to a year to develop tumors. Furthermore, both cell line and patient-derived xenografts can only be established in immunodeficient mice (e.g. athymic, nude, NSG models), which lack both innate and adaptive immune cells. This results in a lack of accuracy in modeling the human tumor microenvironment. Syngeneic mouse models are an alternative strategy, which use mouse tumor cell lines in immunocompetent mice (47); however, these are genetically distinct from human cancers. Though cell line and patient-derived xenografts are limited in reproducing pHGG biology, their high feasibility complements the sophistication of GEM models and allows efficient investigation of CNS penetration and pharmacodynamics of targeted therapies.

### Genetically engineered mouse models

GEM models such as RCAS/TVA, SB, and IUE are very useful for recognizing the molecular events responsible for tumor initiation and growth. While they are technically more complex than PDX, mastery of GEM model generation can produce more time-efficient and more homogeneous survival data than xenografts. Within GEM models, spontaneous generation of tumors using the endogenous loci of the genes better reflects human epigenetic and transcriptional control when compared to overexpression of genes *via* plasmids as in IUE (42). GEM models can provide underlying molecular events during tumor growth in response to specific mutations, as well as allowing the role of the microenvironment to be deciphered (48). However, it is not clear whether the gene changes involved in these models truly mirror the tumor-associated events in human pHGG. GEM tumors are usually composed of cells with specific and homogeneous genetic changes and therefore cannot capture the intratumoral genomic and phenotypic heterogeneity of gliomas. These GEMMs require exogenous promoters to provide upregulated oncogenes, and they determine the geography of malignant transformation through deciding the specific location of plasmid injection/electroporation, both of which illustrate constraints of these models. However, these limitations have largely been addressed with the advent of new advances in germline knock-in mouse models. In neonatal nestin-positive cells throughout the developing murine brain, the mutation *H3.3K27M* has been knocked in to the endogenous *H3F3A* locus, in combination with *TP53* loss and the constitutively active *PDGFRA^V544ins^* mutant, driven by a tamoxifen-inducible Cre recombinase. This model led to spontaneous malignant brain tumor formation, with *H3.3K27M* driving the hindbrain specificity of tumorigenesis, and *PDGFRA* signaling driving pHGG identity.

## Promising therapies

### PDGFR inhibitors

As previously stated, *PDGFRA* alteration has been found more frequently in pediatric HGG compared to adults and is associated with higher pro-tumorigenic potential and worse prognosis (14, 49). Chronic PDGF signaling was found to promote glioma formation in cell culture regardless of mutation profile (3, 50). Therefore, PDGFR pathway alterations in pediatric HGG provide a promising target for PDGFR inhibitors. In addition, Filbin et al. demonstrated that H3K27M-mutant OPC-like cells overexpress *PDGFRA*, potentially opening the door to targeting H3K27M with PDGFR inhibitors (45).

Treatment of thalamic HGG tumor cell cultures from a two-year-old patient with *PDGFRA* amplification with multiple tyrosine kinase inhibitors demonstrated that dasatinib is the most potent inhibitor of cell growth with nanomolar IC50 (14). Dasatinib was less effective in inhibiting proliferation in a pediatric cell culture that had no growth factor receptor amplifications (micromolar IC50). Although a phase II trial found insufficient activity in adult GBM at maximal tolerated dose (51), it is a promising agent in pediatric HGG with PDGF pathway alterations. Dasatinib is well tolerated orally, has moderate blood-brain penetration (52), and demonstrates improved inhibition of PDGFR signaling compared to previous generations of tyrosine kinase inhibitors.

Dasatinib therapy can also synergize with other compounds, such as the mTOR inhibitor everolimus. Although everolimus was being investigated for its blockade of P-gp, we found that this function required micromolar concentrations (too high for clinical use), whereas nanomolar levels of everolimus were able to synergize with dasatinib *in vitro* and prolong survival *in vivo* (41). Additionally, our group published data for four recurrent pHGG patients treated with dasatinib and everolimus that resulted in median progression-free survival (PFS) of 3 months and overall survival (OS) of 8.5 months; therapy was well-tolerated and did not require dose reduction for any patient. These factors indicate that further investigations of dasatinib’s clinical utility as a single agent or combination therapy in *PDGFRA*-altered tumors is warranted (14).

Avapritinib is a *PDGFRA* inhibitor that targets activation loop mutations (e.g. *PDGFRA D842V*) and is more specific to *PDGFRA* and *KIT* than dasatinib (53). Due to its high CNS penetration, avapritinib is a promising option for pHGG therapy and is being investigated in clinical trials (54). Crenolanib, a specific *PDGFRA* and *PDGFRB* inhibitor, demonstrates cytostatic effects in *PDGFRA* amplified and mutated cell lines with no significant effect on cell death (49). Therefore, combination therapy with PDGFR inhibitors may be beneficial for achieving cytotoxic response in pediatric HGG and triggering tumor regression.

There have been less exciting results using other PDGFR inhibitors in clinical trials for pediatric and adult glioma. A phase I trial using imatinib in 84 recurrent pHGG patients revealed significant risk of intratumoral hemorrhage; median PFS and OS were 7 and 11 months, respectively (55). A phase I trial using crenolanib enrolled 32 newly diagnosed and 23 recurrent pHGG patients. For new diagnoses, median PFS and OS were 7 and 12 months; for recurrent tumors, median PFS and OS were 2 and 18 months. No significant differences were seen between groups based on *PDGFRA* alteration, and all outcomes were similar to historical controls (56). Sunitinib was investigated in a phase II trial with 17 recurrent pHGG patients; the study was closed early due to lack of sustained response in all patients (57). The PDGFR inhibitors dovitinib, nintedanib, and tandutinib were tested in phase II adult GBM clinical trials; none showed changes in the survival (58–60).

### EGFR inhibitors

Epidermal growth factor receptor (EGFR) is a member of the ErbB family of tyrosine kinase receptors, which are alpha-helix transmembrane proteins with cytoplasmic tyrosine kinase activity following homodimerization or heterodimerization (61). Many kinds of tumors have activating mutations in exons 18 to 22 of the *EGFR* gene, and the location of these mutations significantly change the effectiveness of EGFR inhibitors (62). *EGFR* amplifications have specifically been found at high frequency in bithalamic and other midline gliomas (13, 18). In cancer cells, inhibition of EGFR with TKIs or monoclonal antibodies can result in decreased cell movement, differentiation, and proliferation. The oral TKIs bind intracellularly and are most effective as monotherapy, while the intravenous monoclonal antibodies operate extracellularly with greater efficacy when used as adjunct therapy (63).

There are many FDA-approved TKIs and monoclonal antibodies for targeting EGFR in various types of cancers. Erlotinib and gefitinib are used for non-small cell lung cancer (NSCLC), and lapatinib is used for HER2-positive breast cancer. Cetuximab is used for colorectal, head and neck cancers, and NSCLC. Primary side effects of TKIs and monoclonal antibodies are diarrhea and acneiform rash, though interstitial lung disease has been reported. Resistance to these EGFR inhibitors arises from mutations in TK, other pathways of cellular proliferation that bypass EGFR, and variations in molecular activity downstream of EGFR (64). Osimertinib showed markedly increased blood-brain barrier penetration in a study with other EGFR inhibitors in brain metastases of NSCLC (65).

EGFR inhibitors have already been investigated in treating pHGG patients. A phase II trial studying gefitinib and radiation enrolled 44 patients and saw six patients with partial response, and three patients who remained progression-free after 36 months. No molecular data was collected, so it is unclear if the latter patients had *EGFR* alterations. Median PFS and OS were 7 and 12 months, similar to controls (66). A phase II study of erlotinib in newly diagnosed pHGG enrolled 41 patients and found median PFS and OS for anaplastic astrocytoma (AA) to be 11 and 15 months, and 6 and 12 months for GBM, respectively (67). These values were not improvements on historical controls. Lapatinib was tested in a phase II trial and enrolled 10 refractory pHGG patients; all cases showed progressive disease (68). Cetuximab was tested in a phase II trial with 25 newly diagnosed DIPG and 20 recurrent non-midline pHGG patients; median PFS was 7 months for DIPG patients and 9 months for non-midline patients, and median OS was 12 and 17 months (69). The PFS for non-midline patients did not meet the 1-year endpoint to continue the trial.

The EGFR inhibitors osimertinib and afatinib were trialed in three patients with bithalamic gliomas (1 grade II, 2 grade III) exhibiting *EGFR* exon 20 insertions (13). All patients exhibited slowly progressive disease on treatment (two alive at publication, 8-19 months after diagnosis; one passed 22 months after diagnosis). Given estimates of median OS for pediatric bithalamic gliomas being around 8 months (70), these results showed promise for moving forward to randomized clinical trials. In the context of adult GBM, afatinib was tested in combination with the alkylating agent temozolomide in a phase I trial; antitumor activity was seen in subsets, but many stopped treatment due to progression or adverse events (71). Dacomitinib was tested in a phase II trial that specifically recruited adult GBM patients with *EGFR* amplification and showed four patients who were progression-free at 6 months, demonstrating activity of the drug (72). Finally, WSD0922 has been investigated for its CNS penetration and is undergoing GBM clinical trials (73, 74).

Recent studies have implicated EGFR as an important modulator of cancer metabolism. In glioblastoma, the *EGFRvIII* mutation (deletion of exons 2-7) was found to regulate expression of hnRNPA1, which in turn spliced a Myc-interacting protein called Max. The splice product Delta Max was found to impart a Myc-dependent glycolytic gene expression pattern to glioblastoma that correlated with poor patient survival (75). Furthermore, kinase-independent EGFR signaling was found to rescue cancer cells from autophagic cell death by maintaining intracellular glucose levels using sodium/glucose cotransporter 1 (SGLT1) (76). As in glioblastoma, EGFR-driven metabolic phenotypes may be responsible for the resistance of pHGG to metabolic drugs such as the imipridones (e.g. ONC201) (77). Although the importance of EGFR in tumorigenesis is undisputed, these studies further highlight how combination therapy using EGFR inhibitors and metabolic modulators may be promising for pHGG.

### FGFR inhibitors

Fibroblast growth factor receptors (FGFR) are a family (FGFR1-4) of tyrosine kinase receptors that have also been implicated in the development and progression of cancers. Aberrant expression of specific FGFR genes is frequently observed in tumors; FGFR1 is the most commonly altered gene of the four in pediatric gliomas, being altered in over a third of cases (78). FGFR2-4 amplification, mutation, and fusion have also been identified and linked to oncogenic activity. These findings indicate that the FGFRs may be a suitable biomarker and therapeutic target (79).

Multi-target TKIs, as well as selective inhibitors of the whole FGFR family, have demonstrated the potential to affect the FGFR pathways involved in tumor development and progression. A recent publication specifically addressing the multi-kinase inhibitor ponatinib CNS penetration by our group showed promising results (52); ponatinib has also demonstrated antiproliferative effects on glioma cells *in vitro* (80). Our group published off-trial use of ponatinib in a case of DIPG with FGFR3 activating mutation, with six months of stable disease before progressing (4-10 months from diagnosis) (78). Ponatinib did not show significant clinical activity in adult GBM (81).

In addition, several FGFR fusions have been discovered that may point to future targeted therapies (82, 83). However, since the FGFR family shares common intracellular signaling pathways with other TKRs, cancer cells may overcome these inhibition therapies by selecting TKR mutants or switching to a parallel signaling pathway (84). Thus, the efficacy of TKR inhibitors in personalized FGFR therapies will be reliant on multi-target TKIs or other drugs with different mechanisms of action.

The pan-FGFR inhibitor erdafitinib has been investigated in a phase I trial for solid tumors, with some glioblastoma patients showing partial responses (85). Both ponatinib and erdafitinib showed favorable CNS penetration based on pharmacokinetic analysis.

### MET and multi-kinase inhibitors

MET is an RTK that contributes to tumor growth and angiogenesis, and its alteration has been identified in recurrent pHGG. Capmatinib is a specific MET inhibitor that was studied in a phase II trial for *MET*-amplified adult GBM and showed no clear activity (86). The MET inhibitor bozitinib (PLB-1001) was tested in 18 recruited pHGG patients; two GBM patients showed partial response, with overall median PFS of 3 months (87).

Several VEGFR inhibitors have been investigated in the hope of combining the antiangiogenic effect with inhibition of common RTK pathways. Tivozanib failed to show antitumor activity in a phase II adult GBM trial, showing limitation of anti-VEGF monotherapy (88). The VEGFR inhibitor sorafenib was investigated in combination with the mTOR inhibitor temsirolimus and the EGFR inhibitor erlotinib in two different phase I/II adult GBM trials, and did not show efficacy in either trial (89, 90). Cediranib showed favorable responses and improved 6-month PFS (26%) in a phase II recurrent adult GBM trial of 31 patients, but did not meet phase III endpoints (91, 92). Apatinib was investigated in a phase II adult glioma trial combined with the topoisomerase inhibitor irinotecan; it enrolled 10 patients and found median PFS to be 8 months and objective response rate (ORR) of 55% (93). Apatinib was further investigated in combination with temozolomide in an adult GBM exploratory study, showing median PFS of 4 months and median OS of 8 months (94).

Cabozantinib, crizotinib, and vandetanib are all multi-tyrosine kinase inhibitors that have been trialed in preclinical pHGG models, targeting MET as well as RET, VEGFR, and ALK, among others. Cabozantinib has been studied preclinically in combination with dasatinib, with promising results (95). Cabozantinib was also investigated in a phase II recurrent adult GBM trial; among the 70 patients who had received prior antiangiogenic therapy, the ORR was 4.3%, described as modest clinical activity (96). Crizotinib was investigated in a phase I trial in combination with dasatinib in 25 recurrent pHGG patients; many dose-limiting toxicities occurred, and no objective radiologic responses were observed (97). A phase I trial using vandetanib in 21 newly diagnosed DIPG patients identified longer PFS in patients with higher VEGF levels before therapy (98). Vandetanib was later investigated in combination with dasatinib in another phase I trial, with median OS of 15 months (99). In addition, vandetanib and everolimus showed promising preclinical results and initial clinical case studies (100). Pazopanib targets several TKIs (among them VEGFR, KIT, PDGFR, and FGFR) to inhibit tumor angiogenesis; it was investigated in a phase II adult glioblastoma trial and did not prolong survival (101). The multi-kinase inhibitor axitinib was investigated in a phase II recurrent adult GBM study that enrolled 55 patients, with one arm adding the alkylating agent lomustine (102). Axitinib resulted in median PFS of 3 months and median OS of 7 months, which were improvements on historical controls. A summary of RTK inhibitors used in preclinical or clinical therapies for pediatric high-grade glioma is listed in **Table 1**.

**Table 1:** Examples of RTK inhibitors used in preclinical or clinical therapies for pediatric high-grade glioma.

Target	Drugs	Observations
PDGFR	Dasatinib Imatinib Avapritinib Crenolanib Sunitinib Dovitinib Nintedanib Tandutinib	No activity in GBM, promising with everolimus (14, 51) Risk of intratumoral hemorrhage (55) Specific to PDGFRA, in clinical trials (53, 54) No significant improvement in clinical trial (56) No significant antitumor activity (57) Phase II GBM trial, no survival change (58) Phase II GBM trial, failed first endpoint (59) Phase II GBM trial, closed after interim analysis (60)
EGFR	Erlotinib Gefitinib Lapatinib Cetuximab Osimertinib Afatinib Dacomitinib WSD0922	Clinical trials showing lack of results (67) Inhibits growth *in vitro*, promising trial results (103, 104) Little single agent activity (68, 105) Unsuccessful phase II clinical trial (69) Cases show slowly progressive disease (13) Cases show slowly progressive disease (13) Activity against EGFR-amplified adult GBM (72) CNS penetrant, undergoing clinical trials (73, 74)
FGFR	Ponatinib Erdafitinib	Showed antiproliferative effect in DIPG cells (80) Response in several glioblastoma patients (85)
MET, multi-kinase	Capmatinib Bozitinib Tivozanib Sorafenib Cediranib Apatinib Cabozantinib Crizotinib Vandetanib Pazopanib Axitinib	No activity in phase II adult GBM trial (86) Achieved partial response in two patients (87) No survival change in phase II adult GBM trial (88) No efficacy in phase I/II adult GBM trials (89, 90) Improved PFS in phase II adult GBM, failed phase III (91, 92) Favorable results in adult GBM trials (93, 94) Modest activity in preclinical and in adult GBM (95, 96) Phase I trial with dasatinib, no activity (97) Trialed alone and with dasatinib or everolimus (98–100) No activity in adult GBM (101) Improved survival in phase II adult GBM trial (102)

## Conclusion and future directions

Due to the numerous RTK alterations in pediatric HGG, as well as potential therapies that already exist, the RTKs are a promising space for targeted therapy. *In vitro* and mouse models have helped discern the behavior of these variant tumors compared with controls, as well as their response to treatment. As modeling pediatric gliomas becomes more advanced, one emerging field is 3D organoid modeling. Recent developments in organoid cultures have harnessed the capacity of human embryonic stem cells (hESCs) or induced pluripotent stem cells (iPSCs) to form 3D aggregates called embryoid bodies. These embryoid bodies can self-organize into a structure that models the cytoarchitecture of organs *in vitro*, from the earliest developmental stages to more mature states (106). These 3D organoids can bridge limitations posed by the 2D cultures and the limitations of *in vivo* models and can serve to accommodate better precision in drug development for adult and pediatric brain tumors.

There are several different 3D organoid models that have been developed for adult brain tumors: glioblastoma organoids (GBOs), neoplastic cerebral organoids, cerebral organoid-glioblastoma cocultures, and bioprinted glioma organoids. GBOs were developed by incorporating minced pieces of GBM specimen with Matrigel in serum-free media (107). GBOs have been shown to have similar histological and transcriptional profiles to their respective patient tumor and recapitulate patient-specific responses and resistance to standard of care therapy (108, 109). A cryopreserved GBO repository has been developed and used to screen a library of 22 compounds that inhibit tumor invasion into surrounding tissue (110). GBOs have also been used to study response to chimeric antigen T (CAR-T) cell immunotherapy in the setting of the *EGFRvIII* variant (111), mTOR inhibitors in *PTEN* loss (112), and STAT inhibitors in Li-Fraumeni syndrome (113). These studies support that GBOs are a reliable tool for developing personalized therapies.

Neoplastic cerebral organoids (neoCORs) are iPSC-derived cerebral organoids that were genetically modified to develop GBM-like tumors by overexpression of oncogenic mutations or downregulation of tumor suppressor genes *via* CRISPR, with green fluorescent protein (GFP) commonly used as a reporter to visualize tumor cell growth and invasion (114, 115). The neoCOR model can be used to study the role of specific mutations in tumor formation and the identification of downstream pathways and specific drug responses. Recently, an *ex vivo* model of medulloblastoma was developed by overexpression of Otx2/c-MYC in cerebellar organoids which showed a disease-specific DNA methylation signature (116). Another recent study generated a malignant glioma model by overexpression of *MEOX2*, inhibition of p53, and loss of PTEN. They showed that MEOX2 increases proliferation in cerebral organoids by activation of the ERK signaling pathway (117).

Cerebral organoid-glioblastoma co-cultures (GLICO) have been developed by co-culturing patient-derived glioblastoma stem cells (GSCs) with cerebral organoids (118, 119). The GLICO model has been used to study the invasive behavior of GFP-marked GSC cell lines, showing stark differences between patients with respect to treatment response. Tumor cells derived from co-culturing also showed a high degree of invasiveness and tumorigenesis when implanted in mice (115). Finally, bioprinted glioma organoids are patient-derived GBM cells that were bioprinted with human umbilical vein endothelial cells and a defined brain extracellular matrix (ECM) (120). This model was shown to replicate patient-specific pathological features and responses to therapy. In summary, brain organoids offer a powerful platform to study the effect of disruptions of central pathways, for identification of novel drug targets, and their resistance mechanisms, and represent a reliable tool for developing personalized therapies.

Another potential strategy for modeling pHGG is the development of patient-specific models in real time. As modeling techniques become validated and efficient, they can be harnessed to generate xenografts that will behave and respond to treatment as the original tumor would. Sequencing can be incorporated to make GEM models for even more accurate and efficient tumor recapitulation. Currently, there is an ongoing clinical trial for medulloblastoma patients that uses biopsy tissue to develop individualized treatment plans (121). Currently, the strategy of characterizing PDXs for therapeutic validation would take weeks to months (27); however, as this technology becomes more efficient, further trials will be able to incorporate mouse models to refine targeted therapies for pHGG.

On the therapeutic side, one direction for RTK-altered pediatric gliomas is co-treatment of multiple TKIs, or one TKI with a synergistic drug to avoid or delay the development of drug resistance (100). As discussed, our group showed that the combination of PDGFRA inhibitor dasatinib with mTOR inhibitor everolimus significantly improved the survival of pediatric high-grade glioma than either treatment alone (41). A phase I/II clinical trial using bevacizumab, irinotecan, and erlotinib in recurrent DIPG enrolled nine patients and resulted in median OS of 13.8 months, compared to radiotherapy-only control median OS of 10 months (122). Due to the crossover between the RTK pathways, the resistance mechanisms that develop to one TKI may depend on another RTK. Akhavan et al. noted that *EGFR* resistance in GBM patients depends on *PDGFRB* deregulation and provided a strong rationale for combination therapy (123). Similarly, Day et al. found that glioblastoma cells treated with EGFR and MET inhibitors can develop resistance *via* FGFR signaling and that simultaneously inhibiting EGFR, FGFR, and MET overcomes the resistance by knocking out more downstream pathways (124). Experimentally, the combination of a PDGFR TKI with another inhibitor targeting either *ERBB3* or *IGF1R* more potently suppressed the growth of GBM cells than either inhibitor alone. Therefore, identifying the RTKs responsible for resistance to one RTK inhibitor may synergistically enhance anti-glioma efficacy.

Another option would be to explore combinations of RTK-targeted therapy and TME-targeted therapy or immunotherapy, such as checkpoint inhibition or CAR-T cell therapy. Both adult and pediatric high-grade gliomas are considered immunologically “cold.” The TME plays an important role in the tumor immune invasion mechanism; tumor cells under TKI treatment may select to respond to the signals from the neighboring stromal cells leading to TME-mediated drug resistance (125). Combining a TKI inhibitor with TME growth factor inhibitors like FGF and SDF1 inhibition could help thwart these resistances. Myeloid-derived suppressor cells (MDSCs) are one of the significant components of the TME; a recent study showed that MDSCs suppress T-cell functions in the glioma TME (37). de Billy et al. combined IGF1R/IR inhibitors with GD2-CAR-T cells and saw increased antitumor activity from the combination in DMG cell lines (126). Exploring the combination of RTK therapy with MDSC inhibition, immune checkpoint inhibition, or CAR-T cell therapy could effectively increase the activation of CD8+ T-cells and change the TME to favor anti-tumor responses.

As we learn more about and experiment with different methods of modeling pediatric glioma, synergism studies between different treatment modalities will be essential in maximizing the efficacy of treatment. Currently, pediatric high-grade gliomas have very few proven effective treatment options. Some possible explanations for the lack of treatment options are the lack of intratumoral heterogeneity in current preclinical models, the limited capacity of small RTK inhibitors to cross the blood-brain barrier (BBB), and specificity/off-target effects of RTK inhibitors. Therefore, it is imperative to develop an individual treatment plan based on each patient’s tumor heterogeneity in terms of specific genetic alteration in combination with enhanced drug delivery approaches. Doing so may provide a much better outcome for pediatric high-grade gliomas, filling this dire need in the clinical setting.

## Author contributions

KS wrote abstract and several sections, synthesized all sections together, and edited manuscript. DM wrote individual sections and helped review. JC, JB, AK, CB, SL, SJ, and RC wrote individual sections. SK helped review. EC initialized process of putting together manuscript. CK and VY oversaw synthesis of manuscript and provided feedback. All authors contributed to the article and approved the submitted version.

## Funding

NIH Grant T32-GM007863 [KS]; NIH Grant T32-CA009676 [DM]; NIH/NINDS Grant R01-NS119231 and R01 NS124607 and Department of Defense Grant CA201129P1, University of Michigan Chad Carr Pediatric Brain Tumor Center, ChadTough Defeat DIPG Foundation, Catching Up With Jack, The Pediatric Brain Tumor Foundation, The Yuvaan Tiwari Foundation, The Morgan Behen Golf Classic, and Michael Miller Memorial Foundation [CK]; ChadTough Defeat DIPG Foundation and Joan and David Evans Prosper Road Foundation [VY].

## Conflict of interest

The authors declare that the research was conducted in the absence of any commercial or financial relationships that could be construed as a potential conflict of interest.

## Publisher’s note

All claims expressed in this article are solely those of the authors and do not necessarily represent those of their affiliated organizations, or those of the publisher, the editors and the reviewers. Any product that may be evaluated in this article, or claim that may be made by its manufacturer, is not guaranteed or endorsed by the publisher.
